# Effects of the glucolipid synthase inhibitor, P4, on functional and phenotypic parameters of murine myeloma cells

**DOI:** 10.1038/sj.bjc.6690792

**Published:** 1999-11

**Authors:** L S Manning, N S Radin

**Affiliations:** Research Centre, Royal Perth Hospital, Perth, Western Australia, 6001, Australia; 350 Sharon Park Dr. – Apt. S5, Menlo Park, CA 94025, USA

**Keywords:** P4 (DL-threo-1-phenyl-2-palmitoylamino-3-pyrrolidino-1-propanol-HCl, multiple myeloma, growth inhibition, apoptosis, phenotypic markers

## Abstract

This study describes the effects of the glucolipid synthase inhibitor P4, (DL-threo-1-phenyl-2-palmitoylamino-3-pyrrolidino-1-propanol), on various functional and phenotypic parameters of 5T33 murine myeloma cells. Cell recovery was reduced by >85% following incubation of the cells for 3 days in the presence of 4 μM P4 (the IC_50_ concentration). Both cytostatic and cytotoxic inhibition was observed with tumour cell metabolic activity and clonogenic potential reduced to 42% and 14% of controls, respectively, and viability reduced to 52%. A dose-dependent increase in cells undergoing apoptosis (from 7% to 26%) was also found. P4 induced a decrease in the number of cells expressing H-2 Class I and CD44, and a large increase in cells expressing H-2 Class II and the IgG_2b_ paraprotein. It did not affect surface expression of CD45 or CD54 (ICAM-1). Based on these alterations in tumour cell growth, adhesion molecule expression and potential immunogenicity, it is anticipated that P4 will provide a novel therapeutic approach for the treatment of multiple myeloma. In addition, given that essentially all tumours rely heavily on overexpressed or abnormal glucosphingolipids for growth, development and metastasis, glucolipid synthase inhibitors may prove to be universally effective anti-cancer agents. © 1999 Cancer Research Campaign


*British Journal of Cancer* (1999) **81**(6), 952–958
© 1999 Cancer Research Campaign
Article no. bjoc.1999.0792

### Effects of the glucolipid synthase inhibitor, P4, on functional and phenotypic parameters of murine myeloma cells

**LS Manning[1] and NS Radin[2]**

[1]Research Centre, Royal Perth Hospital, Perth, Western Australia, 6001, Australia; [2]350 Sharon Park Dr. – Apt. S5, Menlo Park, CA 94025, USA

**Summary** This study describes the effects of the glucolipid synthase inhibitor P4, (DL-threo-1-phenyl-2-palmitoylamino-3-pyrrolidino-1-propanol), on various functional and phenotypic parameters of 5T33 murine myeloma cells. Cell recovery was reduced by >85% following incubation of the cells for 3 days in the presence of 4 μM P4 (the $IC_{50}$ concentration). Both cytostatic and cytotoxic inhibition was observed with tumour cell metabolic activity and clonogenic potential reduced to 42% and 14% of controls, respectively, and viability reduced to 52%. A dose-dependent increase in cells undergoing apoptosis (from 7% to 26%) was also found. P4 induced a decrease in the number of cells expressing H-2 Class I and CD44, and a large increase in cells expressing H-2 Class II and the $IgG_{2b}$ paraprotein. It did not affect surface expression of CD45 or CD54 (ICAM-1). Based on these alterations in tumour cell growth, adhesion molecule expression and potential immunogenicity, it is anticipated that P4 will provide a novel therapeutic approach for the treatment of multiple myeloma. In addition, given that essentially all tumours rely heavily on overexpressed or abnormal glucosphingolipids for growth, development and metastasis, glucolipid synthase inhibitors may prove to be universally effective anti-cancer agents. © 1999 Cancer Research Campaign

**Keywords**: P4 (DL-threo-1-phenyl-2-palmitoylamino-3-pyrrolidino-1-propanol-HCl; multiple myeloma; growth inhibition; apoptosis; phenotypic markers

Cells undergoing developmental, malignant, or viral-induced transformation exhibit marked changes in the relative proportions of their glucosphingolipids (GSLs) (Hakomori, 1986, 1996; Radin, 1994). Tumour cells in particular appear to rely heavily on the accumulation and/or overproduction of GSLs to ensure growth and metastasis, and as a mechanism to evade host immune responses (Li et al, 1995; Hakomori, 1996, 1994; Lavie et al, 1996; Olshefski and Ladisch, 1998). Novel GSLs, differing primarily in the linkages between the different sugar and sialic acid moieties, have also been reported in tumours (Nudelman et al, 1986; Radin, 1994), and a direct correlation between development of multi-drug resistance and increased glucosylceramide synthesis has been demonstrated (Lavie et al, 1996). Given that some or all tumours appear to be dependent on overexpressed or abnormal GSLs for growth, development and metastasis, inhibitors of GSL synthesis have the potential to be universally effective anti-cancer agents.

Studies of a family of GSL synthase inhibitors (‘P-drugs’) representing analogues and homologues of PDMP (D-threo-1-phenyl-2-decanoylamino-3-morpholino-1-propanol) have achieved promising results both in vitro and in vivo as effective anti-neoplastic agents (Inokuchi et al, 1987; Radin and Inokuchi, 1988; Radin et al, 1993; Radin, 1994; Abe et al, 1995). These agents were not only directly cytotoxic to tumour cells but also altered numerous cell functions essential for tumour growth (Radin et al, 1993; Lavie et al, 1996). The effects have been linked to blockage of the early steps in GSL synthesis and to the accumulation of ceramide, which causes marked inhibition of cell growth as well as being an essential early intermediary of both tumour necrosis factor α (TNF-α)- and Fas-mediated apoptosis (Tepper et al, 1995). In vitro, the most active of these inhibitors, P4 (DL-threo-1-phenyl-2-palmitoylamino-3-pyrrolidino-1-propanol), was found to exhibit $IC_{50}$s against human cancer cell lines comparable in effectiveness to several clinically used agents (Abe et al, 1995), suggesting that GSLs play a constitutive role in tumour cell growth and differentiation and thus are potentially useful targets for effective cancer therapy.

Multi-drug resistant (MDR) tumour cells have been shown to be especially sensitive to the growth-inhibiting activity of PDMP (Rosenwald and Pagano, 1994) and the MDR effect has been shown to be reversed by another P-drug (Lavie et al, 1996). In vivo, GSL synthase inhibitors were found to be both well tolerated and to effectively limit tumour growth (Inokuchi et al, 1987, 1990; Nakagawa et al, 1998).

The present study was designed to identify functional and phenotypic alterations induced by the GSL synthase inhibitor, P4, on 5T33 murine myeloma cells. This cell line serves as a well-characterized animal model of human multiple myeloma (Radl et al, 1988; Manning et al, 1992). Multiple myeloma is an incurable B-cell malignancy which accounts for >20% of all human haematological cancers (Greipp, 1992). Although new treatment regimens incorporating bone marrow transplantation achieve 5 times higher complete remission rates and prolonged overall survival compared to conventional therapy, i.e. melphalan and prednisolone, relapse due to residual disease is inevitable, with a median survival of 36 months (Cremer et al, 1997). The effects of

*Received 7 December 1998*
*Revised 6 May 1999*
*Accepted 3 June 1999*

*Correspondence to:* LS Manning

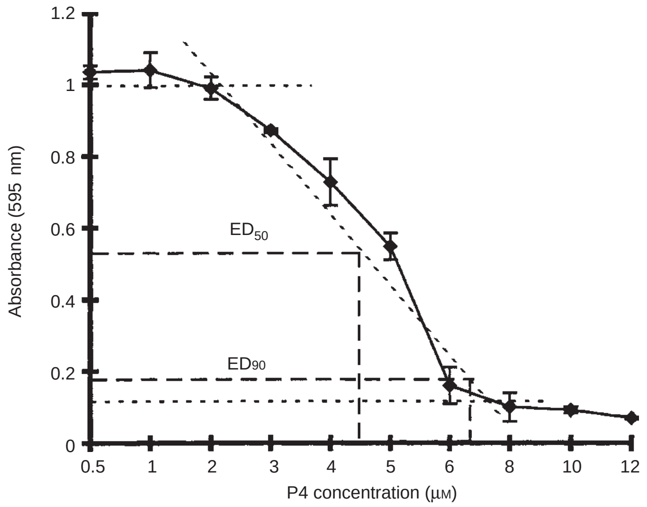

**Figure 1** A representative growth inhibition curve. Cells were incubated with P4 for 4 days and assessed for growth inhibition using the MTT assay as described in Materials and Methods. The data are presented as the mean absorbance at 595 nm (± s.d.) for quadruplicate wells in the presence of increasing concentrations of P4. Short dash lines indicate the linear portion of the curve with the broad dash lines representing the $IC_{50}$ and $IC_{90}$ determinations for this experiment. *n* = 20 experiments

P4 on cell growth, metabolism, clonogenicity and antigen expression were determined in vitro prior to conducting in vivo studies addressing the therapeutic efficacy and immunization potential of this inhibitor in mice. The results reported below demonstrate the cytotoxic and cytostatic potential of P4 in this system.

### MATERIALS AND METHODS

### 5T33 murine multiple myeloma cell line

The cells were maintained in RPMI-1640 medium (Flow Laboratories, Irvine, UK) containing 10% fetal calf serum (FCS; Gibco, Gaithersburg, MD, USA), 2 mM L-glutamine, 100 mM sodium pyruvate, 100 mM non-essential amino acids, penicillin sodium salt (5000 U $ml^{-1}$)/streptomycin sulphate (5 mg $ml^{-1}$) (Trace Biosciences, Castle Hill, NSW, Australia), and 50 μM 2-mercaptoethanol as previously described (Manning et al, 1992). All cells were shown to be free of *Mycoplasma* by the polymerase chain reaction (PCR) assay.

### P4

The inhibitor was from Matreya, Inc. (Pleasant Gap, PA, USA). It was stored as a 5 mM stock solution in sterile, fatty acid-poor bovine serum albumin (BSA)/saline (molar ratio of 5:1, P4:BSA).

### MTT assay

3-(4,5-dimethylthiazo-2-YL)-2,5-diphenyltetrazolium bromide (MTT) was from Sigma Chemical Co. (St Louis, MO, USA) and the metabolic assay was performed according to the method of Mosmann (1983). Briefly, $2.5 \times 10^4$ tumour cells in 0.1 ml were incubated overnight in 96-well, flat-bottomed microtitre plates (Disposable Products Pty Ltd., Adelaide, SA, Australia) and then exposed to 0–12 μM P4 (total volume 0.2 ml) for 4 days. The plates were centrifuged (1000 **g** for 5 min) and 0.1 ml of the supernatant was removed. An aliquot of 30 μl of MTT in phosphate-buffered saline (PBS) (5 mg $ml^{-1}$) was added to each well and the plates were incubated at 37°C for 4–6 h. Extraction buffer (15% sodium dodecyl sulphate (SDS) in 50% v/v sterile water: dimethylformamide; pH 4.7; 0.1 ml) was then added to each well and the plates were incubated overnight at 37°C. Absorbances were determined at 595 nm using a BioRad Model 450 microplate reader (Hercules, CA, USA). They were shown to be directly proportional to cell numbers, achieving an $R^2$ of 0.994 over a range of 250–50 000 5T33 myeloma cells $well^{-1}$ (Manning et al, 1995). The P4 concentrations required to inhibit tumour cell growth by 50% ($EC_{50}$) and by 90% ($EC_{90}$) were determined from the linear portion of the growth inhibition curve (Figure 1).

To assess the recovery of myeloma cell growth following P4 exposure, the cells were first incubated with 0–4 μM P4 in 75 $cm^2$ tissue culture flasks (Corning Costar Corp., Cambridge, MA, USA) for 3 days, then washed and counted by haemacytometer. Viability was checked by trypan blue exclusion. Viable cells ($2.5 \times 10^4$ $well^{-1}$) were then seeded into 96-well microtitre plates and assessed for proliferation by virtue of their metabolic activity after 5 days in inhibitor-free medium, using the MTT assay as described above (Manning et al, 1995).

### Methylcellulose assay

To assess the clonogenic potential of P4-treated cells, the cells were treated as above and diluted to 300 cells $ml^{-1}$ in 2× Dulbecco's modified Eagles medium (DMEM; Flow Labs.) containing 20% FCS. Methylcellulose (Fluka Methocel 65HG, Buchs, Switzerland) was prepared as described by Klinken (1988). Equal volumes of cell suspension and methylcellulose solution (at 37°C) were mixed and 2 ml aliquots were added to triplicate wells of a 24-well plate (Costar). Colonies were counted after incubation at 37°C for 12–14 days.

### Apoptosis detection

Cells were treated with 0–4 μM P4 for 3 days at 37°C, washed, counted and the apoptotic cells detected by virtue of their ability to bind annexin V and exclude propidium iodide (PI). These assays were performed using an R&D Systems Apoptosis Detection Kit (Minneapolis, MN, USA) following the manufacturer's instructions. Cells were analysed using a Coulter EPICS XL-MCL (Flow Cytometry Section, Division of Laboratory Medicine, Royal Perth Hospital) with gates set at $10^1$ log fluorescence for PI and $10^2$ log fluorescence for fluorescein isothiocyanate (FITC)–annexin V. Five thousand cells were counted for each sample. The data are presented as the percent viable (unstained) cells, cells undergoing necrosis (stained with both FITC–annexin V and PI) or cells undergoing apoptosis (stained with FITC–annexin V only).

### Phenotypic analysis

The effects of P4 on cell surface expression of differentiation antigens (H-2 Class I, H-2 Class II and CD45), adhesion molecules (CD44 and CD54) and the $IgG_{2b}$ paraprotein were examined by incubating the cells with 0–4 μM P4 for 3 days, and assessing surface antigen expression using optimum concentrations (determined by titration) of the following antibodies: mouse anti-mouse H-2 pan Class I (kindly provided by Dr P Price, Department of

© 1999 Cancer Research Campaign

**Table 1** Effects of P4 on 5T33 myeloma cell functional parameters

| Functional parameter | P4 concentration (μM) 0 | 0.5/1[a] | 2 | 3 | 4 |
|---|---|---|---|---|---|
| | mean ± s.e.m. (% of 0 control) | | | | |
| I. Cell recovery[b] × $10^6$ cells | 14.9±1.0 (100%) | 11.7±0.7* (78.6%) | 8.5±0.5* (56.8%) | 5.0±0.6* (33.5%) | 2.0±0.6* (13.3%) |
| II. Cell proliferation[c] fold change in cell #s | 5.4±0.4 (100%) | 3.9±0.3* (72.2%) | 3.0±0.3* (55.6%) | 1.7±0.3* (31.5%) | –0.4±0.4* (–92%) |
| III. Viability[d] % viable cells | 93.9±0.7 (100%) | 91.1±1.4 (96.9%) | 88.9±1.6 (94.7%) | 76.1±3.4* (81.0%) | 52.1±6.6* (55.3%) |
| IV. Regrowth potential[e] | 1.18±0.07 (100%) | 1.00±0.08 (93.9%) | 0.85±0.08* (78.8%) | 0.65±0.08* (64.1%) | 0.44±0.04* (41.7%) |
| V. Clonogenic potential[f] # of clones $well^{-1}$ | 43.4±5.2 (100%) | 26.1±5.1* (60.1%) | 20.0±3.1* (45.9%) | 13.2±3.1* (27.6%) | 5.4±2.4* (13.9%) |

Cells were incubated for 3 days in the presence of 0–4 μM P4, washed and assessed for functional activity. N = 10 experiments for each parameter. [a]No difference was observed for cells treated with 0.5 or 1 μM P4, so the data have been pooled. [b]Cell recovery was determined by cell counting using a haemacytometer. The data are presented as the mean number of viable cells recovered ± s.e.m. The values in parentheses represent the % cell recovery compared to untreated cells. [c]Cell proliferation during the 3-day incubation period was calculated from the starting cell number $flask^{-1}$, i.e. $3 \times 10^6$ cells. The data are presented as the mean fold change in cell numbers ± s.e.m. The values in parentheses represent the % cell proliferation compared to untreated cells. [d]Cell viability was determined by trypan blue exclusion. The data are presented as the mean % viable cells ± s.e.m. The values in parentheses represent the % viable cells compared to untreated cells. [e]The regrowth potential of P4-treated cells was determined using the MTT assay as described in Materials and Methods. The data are presented as the mean absorbance at 595 nm ± s.e.m. The values in parentheses represent the % regrowth potential compared to untreated cells. [f]The clonogenic potential of P4-treated cells was determined by colony formation in methylcellulose as described in Materials and Methods. The data are presented as the mean number of colonies $well^{-1}$ ± s.e.m. The values in parentheses represent the % number of colonies compared to untreated cells. The clonogenic potential of untreated 5T33 myeloma cells ranged from 10.8 to 18.2%. *$P < 0.001$ compared to untreated cells.

Immunology, Royal Perth Hospital, Perth, WA, Australia), rat anti-mouse H-2 I-A and rat anti-mouse CD45 (both from Serotec Ltd., Oxford, UK), rat anti-mouse CD44 and rat anti-mouse ICAM-1 (CD54) (both from R&D Systems), and rat anti-mouse $IgG_{2b}$ (Biosource Int., Camarillo, CA, USA). Antibody binding was detected using a biotinylated goat $F(ab')_2$ anti-rat Ig followed by a streptavidin–fluorescein conjugate (both obtained from Biosource). For each sample, 10 000 cells were analysed using the Coulter EPICS.

### Statistical analysis

Data were analysed using the two-tailed, paired Student's *t*-test and are presented as the mean ± standard deviation (s.d.) or standard error of the mean (s.e.m.).

### RESULTS

### Inhibition of 5T33 myeloma cell growth by P4

P4 effectively inhibited cell growth, demonstrating an $IC_{50}$ of 3.9 ± 0.3 μM for 20 experiments. A representative growth inhibition curve is shown in Figure 1. Growth inhibition of the cells occurred over a small P4 concentration range, with the $IC_{90}$ achieved at only slightly higher concentration than the $IC_{50}$, i.e. 7.1 ± 0.3 μM.

### Kinetics of P4-induced growth inhibition

Significant growth inhibition was achieved by incubating cells in the presence of only 1 μM P4 (Figure 2). Viable cell recovery was reduced from $18 \pm 2 \times 10^6$ for untreated cells to $1.5 \pm 0.1 \times 10^6$ for cells exposed to 4 μM P4 for 3 days (92% reduction). These remaining cells represent only 50% of the starting cell number (i.e. $3 \times 10^6$ $flask^{-1}$) suggesting that a large proportion of the tumour cells are actually being destroyed by P4. As day 4 control cultures demonstrated reduced cell recovery as compared to day 3, probably due to nutrient and GSL depletion, all remaining experiments were conducted using cells exposed to P4 for 3 days.

### Effects of P4 on 5T33 myeloma cell functional activities

Table 1 depicts the effects of P4 on various functional parameters essential for tumour growth and development. Inhibition of all parameters tested was found to be dose-dependent with the $IC_{50}$ concentration (i.e. 4 μM) reducing functional activities by 45–88%. Both cytostatic and cytotoxic growth inhibition were observed. Significant cytostatic inhibition was observed at the lowest concentration of P4 tested (0.5 μM) with cell recovery and proliferation reduced by 21% and 28% respectively ($P < 0.001$). In contrast, significant loss of cell viability, indicative of cytotoxic activity, was only achieved at the higher concentrations of P4, 3–4 μM ($P < 0.001$). Exposure of the cells to 4 μM P4 for 3 days reduced cell viability by 45%. The same concentration decreased cell recovery by 87% ($P < 0.001$). As shown in Figure 2, cell recovery following 4 μM P4 treatment was actually 33% less than the starting cell number (2 vs $3 \times 10^6$ cells; Table 1) resulting in a negative proliferation index for these cultures (–0.4). These findings further support direct tumour cell lysis by P4 in addition to its cytostatic activity.

Using metabolic activity as a measure of cell numbers, P4 exposure was found to significantly decrease myeloma cell growth with 4 μM P4 reducing metabolic activity by 58% compared to

 *© 1999 Cancer Research Campaign*

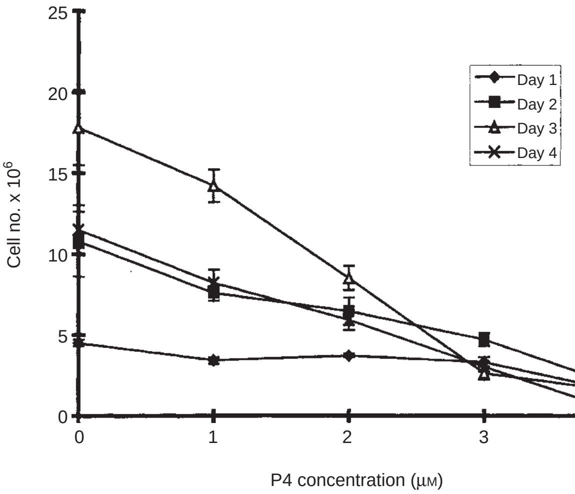

**Figure 2** Kinetics of P4-induced growth inhibition on 5T33 myeloma cell survival. Cells were incubated in the presence of 0–4 μM P4 for 1–4 days and assessed for viable cell recovery as described in Materials and Methods. The data are presented as the mean viable cells × $10^6$ (± s.d.) for duplicate flasks for 3 experiments. Starting cell concentration = 3 × $10^6$ cells $flask^{-1}$

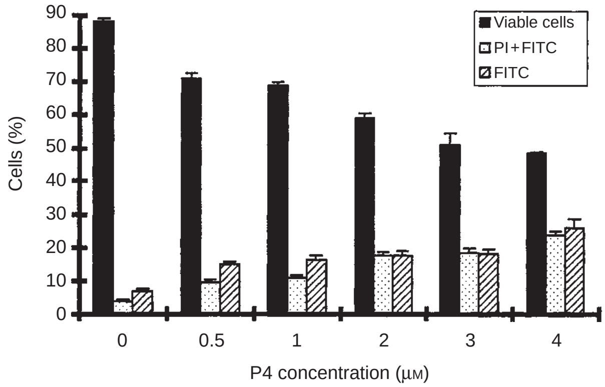

**Figure 3** Dose-dependent induction of apoptosis in 5T33 myeloma cells by P4. Cells were incubated in the presence of 0–4 μM P4 for 3 days, washed and assessed for apoptosis as described in Materials and Methods. The data are presented as the percentage (± s.e.m.) of viable cells (■); necrotic cells (⬚) and cells undergoing apoptosis (▨). *n* = 7 experiments. $P < 0.01$ for all three cell populations compared to untreated cells untreated cells (Table 1; $P < 0.001$). The clonogenic potential of the cells was even more sensitive to the inhibitory effects of P4 with exposure to 0.5 μM P4 inducing a 40% decrease in clonogenic potential (from 43 ± 5 to 26 ± 5 colonies $well^{-1}$; $P < 0.001$). The $IC_{50}$ concentration (4 μM) of P4 inhibited colony formation by 86% ($P < 0.001$).

### Induction of apoptosis by P4

The dose-dependent loss of viability observed for P4-treated cells was found to be due both to necrosis and to apoptosis (Figure 3). At lower concentrations of P4 (0.5–1 μM), the increase in necrotic cells alone (from 4 ± 0.4% to 10 ± 1%) did not significantly decrease cell viability, which supports the viability results shown in Table 1. However, 15 ± 1% of the cells treated with 0.5 μM P4 were found to be undergoing apoptosis (Figure 3). This represents a population of ‘non-viable’ cells that would not be detected by the trypan blue assay, suggesting that even at low P4 concentrations, a significant number of the cells are programmed for cell death. Cell viability was reduced from 88 ± 1% to 48 ± 1% following exposure to 4 μM P4 for 3 days with 26 ± 2% of the non-viable cells undergoing apoptosis (Figure 3; $P < 0.001$).

### Effects of P4 on surface antigen expression

The effects of P4 treatment on expression of cell surface antigens involved in differentiation, adhesion and immunogenicity were evaluated as potential markers of functional alterations. Both the Class I and Class II antigen presentation molecules demonstrated dose-dependent alterations in cell surface expression following incubation with P4 (Table 2). Myeloma cell exposure to as little as 2 μM P4 was sufficient to reduce the number of cells expressing the Class I antigen from 95 ± 1% to 87 ± 1% ($P < 0.001$), with 4 μM P4 decreasing this number even further. Surface expression

**Table 2** Effects of P4 on 5T33 myeloma cell phenotypic parameters

| | **P4 concentration (μM)** | | | | |
|---|---|---|---|---|---|
| | **0** | **0.5/1[a]** | **2** | **3** | **4** |
| **Phenotypic parameter** | **mean % fluorescing cells ± s.e.m.** | | | | |
| I. Differentiation Ags | | | | | |
| H-2 Class I | 94.5±0.7 | 90.9±1.4 | 87.4±1.4* | 77.5±2.3* | 61.4±3.7* |
| H-2 Class II | 1.7±0.4 | 3.0±0.9 | 6.0±1.2* | 8.6±2.2* | 14.1±2.7* |
| CD45 | 89.9±1.4 | 90.6±1.0 | 88.0±1.8 | 88.5±1.8 | 89.9±2.0 |
| II. Adhesion molecules | | | | | |
| CD44 | 89.5±1.6 | 80.0±2.1 | 72.8±3.0* | 59.5±3.7* | 38.0±2.3* |
| CD54 | 92.9±0.9 | 90.9±1.1 | 91.7±1.0 | 89.6±4.0 | 93.3±0.3 |
| III. Paraprotein | | | | | |
| $IgG_{2b}$ | 5.6±0.8 | 8.2±0.9 | 13.8±1.0* | 18.8±2.2* | 26.9±3.6* |

Cells were incubated for 3 days in the presence of 0–4 μM P4, washed and assessed for cell surface antigen expression. The data are presented as the mean % fluorescing cells ± s.e.m. for 6–10 experiments for each antigen. Fluorescence was detected using the Coulter EPICS flow cytometer and 10 000 cells (events) were analysed for each sample. [a]No difference was observed for cells treated with 0.5 or 1 μM P4 so the data have been pooled. *$P < 0.001$ compared to untreated cells.

© 1999 Cancer Research Campaign of Class I was observed on only 61 ± 4% of cells following treatment with 4 μM P4 (Table 2; $P < 0.001$). In complete contrast, Class II antigen expression was markedly increased by the inhibitor, with 4 μM P4 increasing expression almost tenfold (from 2 ± 0.4% to 14 ± 3%; $P < 0.001$). Approximately 90% of the cells were found to express the differentiation antigen, CD45, which is a marker for primitive to immature plasma cells/myeloma cells (Schneider et al, 1997), and this expression was not altered by P4 exposure regardless of the inhibitor concentration (Table 2).

CD44 has been shown to be an important adhesion molecule for myeloma cell homing to the bone marrow and binding to bone marrow stromal cells, whereas CD54 (ICAM-1) appears to play only a minor role in this aspect of disease development (Okada and Hawley, 1995). Of interest, P4 was found to induce a striking dose-dependent decrease in CD44 expression without altering CD54 expression (Table 2). Whereas 90 ± 2% of untreated cells expressed CD44, only 38 ± 2% of cells exposed to 4 μM P4 maintained surface expression of this antigen ($P < 0.001$). ICAM-1 (CD54) expression, on the other hand, was observed on > 90% of all the cells tested regardless of P4 exposure. These results imply an important role for GSLs in CD44 function.

The myeloma paraprotein has been shown to be a highly immunogenic molecule capable of inducing therapeutically effective anti-idiotypic immune responses in both mice and humans (Croese et al, 1991*a*, 1991*b*; Kwak et al, 1995). P4 exposure was found to induce a large, dose-dependent increase in the number of cells expressing $IgG_{2b}$ (Table 2). Only 5.6 ± 0.8% of untreated cells expressed the paraprotein on their surface compared to 26.9 ± 3.6% of cells exposed to 4 μM P4 for 3 days ($P < 0.001$).

### DISCUSSION

The pattern of growth inhibition observed for P4 on the 5T33 murine myeloma cells used in this study (Figure 1) was similar to those previously reported for this and other 'P-drugs' against five types of human malignancy (Abe et al, 1995). The 3.9 μM $IC_{50}$ P4 concentration is comparable to those reported for the human cell lines ($IC_{50}$s of 0.7–2.6 μM). The $IC_{90}$ P4 concentration was less than twice the $IC_{50}$ (i.e. 7.1 μM). P4-induced growth inhibition of both the human and murine cell lines was found to occur over a very small concentration range, as indicated by the steep slopes of the growth inhibition curves (Abe et al, 1995; Figure 1). The uniformity of inhibitor activity observed against the various tumour types provides additional evidence that GSLs play a constitutive role in tumour cell growth and differentiation. It also suggests that GSL synthase inhibitors may have a wider therapeutic application than currently used cytotoxic drugs. The fact that P-drugs have been shown to be effective against MDR tumour cells (Rosenwald and Pagano, 1994; Lavie et al, 1996) is of particular interest given that disease relapse due to the emergence of drug-resistant cells remains the leading cause of death of patients with many types of tumours, including multiple myeloma. Because the 5T33 murine myeloma cells do not express surface P-glycoprotein 170 (P-gp170; data not shown), human myeloma cells of known MDR phenotype will be used to address this important issue.

Several tumour cell functions were found to be markedly down-regulated at P4 concentrations (0.5–1 μM), which induced only minimal cytotoxicity (Table 1). A significant reduction in cell recovery following 3 day incubation with P4 was observed at the lowest concentration of P4 tested (0.5 μM), apparently a reflection of a reduction in both cell proliferation and metabolic activity. Clonogenic potential was also significantly reduced by exposure to low-dose P4, whereas tumour cell viability was not altered under these conditions (Table 1). Significant loss of tumour cell viability, as determined by trypan blue uptake, was only observed following incubation with 3–4 μM P4 (Table 1). In fact, cell recovery was reduced by > 33% of starting cell numbers under these conditions suggesting direct cytolysis of a substantial number of tumour cells by high-dose P4. This dose-dependent distinction between reduced cell growth and induced cell death by GSL synthase inhibitors has been previously reported (Inokuchi et al, 1990; Rosenwald and Pagano, 1994; Abe et al, 1995). It is likely that cells engage in a 'yin and yang' competition, in which the proliferative effects of glucosylceramide oppose the repressive apoptotic effects of its precursor, ceramide.

The impaired growth and clonogenic potential we observed in treated cells even after the cells were transferred to normal medium, may be the result of retention of P4 by the cells. A test with Madin–Darby canine kidney cells showed, by chromatographic analysis and enzyme assay, that P4 does not readily leave P4-treated cells, even when the cells are homogenized and washed (Abe et al, 1996). This prolonged action would have the advantage of maintaining viable, non-replicating tumour cells as targets for immune activation. It also suggests that frequent dosing of P4 in a cancer-bearing patient may not be necessary to maintain therapeutic efficacy.

This study identified a dose-dependent increase in cells undergoing apoptosis; which may play a significant role in both the reduced tumour cell recovery and viability observed following P4 exposure (Table 1 and Figure 3). At the lower concentrations of P4 (0.5–1 μM), the increase in necrotic cells alone was not sufficient to significantly reduce tumour cell viability (95% to 89%), which correlates with the previous trypan blue exclusion results (Table 1). However, a substantial increase in cells undergoing apoptosis was identified by flow cytometry (from 7% to 15%; Figure 3). Significant cytotoxicity was observed following cell exposure to 4 μM P4 for 3 days, with necrotic cells increasing to > 23% of the tumour cell population (Figure 3). An additional 26% of the cells were programmed for cell death, i.e. undergoing apoptosis, which indirectly supports the concept of inhibitor-induced accumulation of ceramide (Figure 3). Abe et al (1995) clearly showed ceramide accumulation at this concentration of P4. Overall, the population defined as 'viable' by this assay was reduced to 55% of control cells under these conditions. These results suggest that in addition to being directly toxic to tumour cells, high-dose glucolipid synthase inhibitor therapy effectively limits the growth potential of a substantial number of the 'surviving' cells.

Cell surface GSLs have been implicated as essential molecules in tumour cell attachment, immune evasion and metastatic spread (Inokuchi et al, 1990; Hakomori, 1994; Kannagi, 1997). In this study, alterations in several cell surface molecules were readily identified following P4 exposure. The proportion of cells expressing surface H-2 Class I antigen was reduced by P4 in a dose-dependent manner (Table 2). Several inhibitor-induced alterations may be involved in this effect, including disruption of receptor assembly and transport, of anchoring and presentation on the cell surface, or of β-2-microglobulin (β-2M) production, which is essential for Class I antigen expression (Karre, 1993; Radin et al, 1993; Rosenwald and Pagano, 1994). Although the loss of Class I positive tumour cells may reduce CD8+ cytotoxic T-cell (CTL) recognition and activation, susceptibility to killing by

 © 1999 Cancer Research Campaign natural killer (NK) cells should be enhanced in this setting since Class I expression is inhibitory to NK cell function (Karre, 1993). The immunosurveillance role of NK cells is thought to be particularly important in controlling haematological malignancies and circulating metastatic foci (Karre, 1993).

In contrast to the effect of P4 on Class I expression, the number of cells expressing H-2 Class II was greatly increased following P4 exposure (Table 2). Increased expression of Class II has been shown to be a positive prognostic factor in several tumour systems, resulting in effective immune activation and subsequent tumour regression in some cases (Baskar et al, 1995; Cabrera et al, 1995; Armstrong et al, 1997). Experiments are currently underway that address P4-induced activation of a tumour-specific immune response in 5T33 myeloma-bearing mice.

The common leucocyte antigen, CD45, which has been shown to be most highly expressed on primitive and immature plasma cells and myeloma cells (Schneider et al, 1997), was not affected by P4 exposure. Taken together, the results suggest that tumour cells utilize GSLs extensively, but selectively, to maximize survival.

In terms of adhesion molecules, Okada and Hawley (1995) have demonstrated that murine myeloma cells, like human myeloma cells, express surface CD44, ICAM-1 (CD54) and VLA-4. In addition, CD44 and VLA-4 were shown to be involved in myeloma cell binding to bone marrow stromal cells (i.e. endothelial cells, fibroblasts and adipocytes) with ICAM-1 only partially involved in binding to bone marrow-derived macrophages. The present study revealed a marked, P4-dependent decrease in cells expressing CD44 (Table 2). This response is to be expected based on the observation that CD44 is closely associated with GSL-rich plasma membrane domains (Ilangumaran et al, 1998), and may have important implications for myeloma development. Hyaluronan, the ligand for CD44, is found in high concentration in bone marrow (Aruffo et al, 1990) and is known to play an important role in tumour homing and metastasis (Sherman et al, 1994). It is possible that the loss of CD44 expression would not only interfere with tumour seeding in the appropriate micro-environment, i.e. the bone marrow, but would also relegate tumour cells to the circulation and lymphatics, thereby increasing their exposure to the immune system and allowing effective immune activation prior to tumour establishment. Experiments are currently underway in which mice and/or tumour cells are treated with P4 prior to tumour cell inoculation to investigate inhibitor effects on both tumour development and immune induction in vivo.

The myeloma paraprotein has been shown to be a highly immunogenic molecule capable of inducing both tumour-specific anti-idiotypic antibodies as well as activating idiotypic-specific T-cells (Croese et al, 1991*a*, 1991*b*; Kwak et al, 1992, 1995; Dabadghao et al, 1998). Here, we report a significant, dose-dependent increase in 5T33 myeloma cells expressing surface $IgG_{2b}$ paraprotein (up to 27%; Table 2). Increased expression of this highly immunogenic antigen may enhance immune recognition with subsequent activation of a protective tumour-specific immune response.

The results of this and many other studies strongly suggest that GSL synthase inhibitors have the potential to be a universally effective form of cancer treatment. Given that current therapies are not curative for the majority of human malignancies, alternative forms of treatment need to be developed to improve the prognosis of patients with cancer. It is anticipated that continued investigations of both the therapeutic and immunization potential of GSL synthase inhibitors will clarify the efficacy of these inhibitors as novel therapeutic agents and lend themselves to the development of more effective treatment for patients with cancer.

### ACKNOWLEDGEMENTS

This work was supported by the Royal Perth Hospital Medical Research Foundation.

### REFERENCES

Abe A, Radin NS, Shayman JA, Wotring LL, Zipkin RE, Sivakumar R, Ruggieri JM, Carson KG and Ganem B (1995) Structural and stereochemical studies of potent inhibitors of glucosylceramide synthase and tumour cell growth. *J Lipid Res* **36**: 611–621

Abe A, Radin NS and Shayman JA (1996) Induction of glucosylceramide synthase by synthase inhibitors and ceramide. *Biochim Biophys Acta* **1299**: 333–341

Armstrong TD, Clements VK, Martin BK, Ting JP-Y and Ostrand-Rosenberg S (1997) Major histocompatibility complex Class II-transfected tumour cells present endogenous antigen and are potent inducers of tumour-specific immunity. *Proc Natl Acad Sci USA* **94**: 6886–6891

Aruffo A, Stamenkovic I, Melnick M, Underhill CB and Seed B (1990) CD44 is the principal cell surface receptor for hyaluronate. *Cell* **61**: 1303–1313

Baskar S, Glimcher L, Nabavi N, Jones RT and Ostrand-Rosenberg S (1995) Major histocompatibility complex Class II+, B7-1+ tumour cells are potent vaccines for stimulating tumour rejection in tumour bearing mice. *J Exp Med* **181**: 619–629

Cabrera T, Ruiz-Cabello F and Garrido F (1995) Biological implications of HLA DR expression in tumours. *Scand J Immunol* **41**: 398–406

Cremer FW, Kiel K, Wallmeier M, Goldschmidt H and Moos M (1997) A quantitative PCR assay for the detection of low amounts of malignant cells in multiple myeloma. *Ann Oncol* **8**: 633–636

Croese JW, Vissinga CS, Boersma WJA and Radl J (1991*a*) Immune regulation of mouse 5T2 multiple myeloma. I. Immune response to 5T2 MM idiotype. *Neoplasma* **38**: 457–466

Croese JW, Van Den Enden-Vieveen MHM, Radl J (1991*b*) Immune regulation of mouse 5T2 multiple myeloma. II. Immunological treatment of 5T2 MM residual disease. *Neoplasma* **38**: 467–474

Dabadghao S, Bergenbrant S, Anton D, Wen H, Holm G and Yi Q (1998) Anti-idiotypic T-cell activation in MM induced by M-component fragments presented by dendritic cells. *Br J Haematol* **100**: 647–654

Greipp R (1992) Advances in the diagnosis and management of myeloma. *Semin Hematol* **29**: 24–45, 1992.

Hakomori S (1986) Glycosphingolipids. *Sci Am* **254**: 44–53

Hakomori S (1994) Role of gangliosides in tumour progression. *Prog Brain Res* **101**: 241–250

Hakomori S (1996) Tumour malignancy defined by aberrant glycosylation and sphingo(glyco)lipid metabolism. *Cancer Res* **56**: 5309–5318

Ilangumaran S, Briol A and Hoessli DC (1998) CD44 selectively associates with active Src family protein tyrosine kinases Lck & Fyn in GSL-rich plasma membrane domains of human peripheral blood lymphocytes. *Blood* **91**: 3910–3908

Inokuchi J, Mason I and Radin NS (1987) Antitumor activity via inhibition of glucosphingolipid biosynthesis. *Cancer Lett* **38**: 23–30

Inokuchi J, Jimbo M, Momosaki K, Shimeno H, Nagamatsu A and Radin NS (1990) Inhibition of experimental metastasis of murine Lewis lung carcinoma by an inhibitor of glucosylceramide synthase and its possible mechanism of action. *Cancer Res* **50**: 6731–6737

Kannagi R (1997) Carbohydrate-mediated cell adhesion involved in hematogenous metastasis of cancer. *Glycoconjugate J* **14**: 577–584

Karre K (1993) Natural killer cells and the major histocompatibility complex Class I pathway of peptide presentation. *Semin Immunol* **5**: 127–145

Klinken SP (1988) Erythroproliferation in vitro can be induced by abl, fes, src, ras, bas, raf, faf/myc, erb B and cbl oncogenes but not by myc, myb, and fos. *Oncogene Res* **3**: 187–192

Kwak LW, Campbell MJ, Czerwinski DK, Hart S, Miller RA and Levy R (1992) Induction of immune responses in patients with B-cell lymphoma against the surface-immunoglobulin idiotype expressed by their tumors. *N Engl J Med* **327**: 1209–1215

Kwak LW, Taub DD, Duffey PL, Bensinger WI, Bryant EM, Reynolds CW and Longo DL (1995) Transfer of myeloma idiotype-specific immunity from an actively immunised marrow donor. *Lancet* **345**: 1016–1020

© 1999 Cancer Research Campaign

Lavie Y, Cao H, Bursten SL, Giuliano AE and Cabot MC (1996) Accumulation of glucosylceramides in multi-drug resistant cancer cells. *J Biol Chem* **271**: 19530–19536

Li R, Villacreses N and Ladisch S (1995) Human tumor gangliosides inhibit murine immune responses in vivo. *Cancer Res* **55**: 211–214

Manning LS, Berger JD, O'Donoghue HL, Sheridan G, Claringbold PG and Turner JH (1992) A model of multiple myeloma: culture of 5T33 murine myeloma cells and evaluation of tumorigenicity in the C57BL/KaLwRij mouse. *Br J Cancer* **66**: 1088–1093

Manning LS, Chamberlain NL, Leahy MF and Cordingley FT (1995) Assessment of the therapeutic potential of cytokines, cytotoxic drugs and effector cell populations for the treatment of multiple myeloma using the 5T33 murine myeloma model. *Immunol Cell Biol* **73**: 326–332

Mosmann T (1983) Rapid colorimetric assay for cellular growth and survival: application to proliferation and cytotoxicity assays. *J Immunol Meth* **65**: 55–63

Nakagawa R, Motoki K, Ueno H, Iijima R, Nakamura H, Kobayashi E, Shimosaka A and Koezuka Y (1998) Treatment of hepatic metastasis of the colon26 adenocarcinoma with an anti-galactosylceramide, KRN7000. *Cancer Res* **58**: 1202–1207

Nudelman E, Levery SB, Kaizu T and Hakomori S (1986) Novel fucolipids of human adenocarcinoma: characterisation of the major $Le^y$ antigen of human carcinoma as trifucosylnonaosyl $Le^y$ glycolipid. *J Biol Chem* **261**: 11247–11253

Okada T and Hawley RG (1995) Adhesion molecules involved in binding of murine myeloma cells to bone marrow stromal elements. *Int J Cancer* **63**: 823–830

Olshefski R and Ladisch SJ (1998) Synthesis, shedding, and intercellular transfer of human medulloblastoma gangliosides: abrogation by a new inhibitor of GlcCer synthase. *Neurochem* **70**: 467–472

Radin NS (1994) Rationales for cancer chemotherapy with PDMP, a specific inhibitor of glucosylceramide synthase. *Mol Chem Neuropathol* **21**: 111–127

Radin NS and Inokuchi, J-I (1988) Glucosphingolipids as sites of action in the chemotherapy of cancer. *Biochem Pharmacol* **37**: 2879–2889

Radin NS, Shayman JA and Inokuchi J-I (1993) Metabolic and functional effects of inhibiting glucosylceramide synthesis with PDMP and other substances. *Adv Lipid Res* **26**: 183–213

Radl J, Croese JW, Zurcher C, Van Den Enden-Vieveen MHM and De Leeuw AM (1988) Multiple myeloma: animal model of human disease. *Am J Pathol* **132**: 593–597

Rosenwald AG and Pagano RE (1994) Effects of the glucosphingolipid synthesis inhibitor, PDMP, on lysosomes in cultured cells. *J Lipid Res* **35**: 1232–1240

Schneider U, van Lessen A, Huhn D and Serke S (1997) Two subsets of peripheral blood plasma cells defined by differential expression of CD45 antigen. *Br J Haematol* **97**: 56–64

Sherman L, Sleeman J, Herrlich P and Ponta H (1994) Hyaluronate receptors: key players in growth, differentiation, migration and tumor progression. *Curr Opin Cell Biol* **6**: 726–733

Tepper CG, Jayadev S, Liu B, Bielawska A, Wolff R, Yonehara S, Hannun YA and Seldin MF (1995) Role of ceramide as an endogenous mediator of fas-induced-cytotoxicity. *Proc Natl Acad Sci USA* **92**: 8443–8447

*© 1999 Cancer Research Campaign*
